# Anti-Citrullinated Peptide Antibody Expression and Its Association with Clinical Features and Outcomes in Patients with Antineutrophil Cytoplasmic Antibody-Associated Vasculitis

**DOI:** 10.3390/medicina58040558

**Published:** 2022-04-18

**Authors:** Sung Soo Ahn, Jung Yoon Pyo, Jasong Jungsik Song, Yong-Beom Park, Sang-Won Lee

**Affiliations:** 1 Department of Internal Medicine, Yongin Severance Hospital, Yonsei University College of Medicine, Yongin 16995, Korea; saneth@yuhs.ac; 2 Division of Rheumatology, Department of Internal Medicine, Yonsei University College of Medicine, Seoul 03722, Korea; jyp@yuhs.ac (J.Y.P.); jsksong@yuhs.ac (J.J.S.); yongbpark@yuhs.ac (Y.-B.P.); 3 Institute for Immunology and Immunological Diseases, Yonsei University College of Medicine, Seoul 03722, Korea

**Keywords:** antineutrophil cytoplasmic antibody, anti-citrullinated peptide antibody, feature, outcome, vasculitis

## Abstract

*Background and objectives*: Anti-citrullinated peptide antibody (ACPA), a characteristic antibody detected in rheumatoid arthritis, could be linked to antineutrophil cytoplasmic antibody (ANCA)-associated vasculitis (AAV) via the formation of neutrophil extracellular traps. We investigated the rate of ACPA positivity in patients with AAV and evaluated the association of ACPAs with their clinical features and outcomes. *Materials and Methods*: A total of 168 AAV patients with both ACPA and ANCA results at diagnosis were identified. Clinical and laboratory variables, including the disease-specific indices of Birmingham Vasculitis Activity Score (BVAS) and Five-Factor Score (FFS), were investigated. All-cause mortality, relapse, and end-stage renal disease, as well as interstitial lung disease (ILD) were evaluated as outcomes of the patients, and the Kaplan–Meier survival analysis was used to compare the event-free survival rates of the groups. *Results:* Fifteen (8.9%) and 135 (80.4%) patients were positive for ACPA and ANCA, respectively. There were no significant differences in the baseline variables of ACPA-negative and ACPA-positive patients. The absolute titre of ACPAs also did not significantly correlate with BVAS, FFS, erythrocyte sedimentation rate, or C-reactive protein. In addition, there was no difference noted regarding overall, relapse-free, and ESRD-free survival rates between ACPA-negative and ACPA-positive AAV patients. However, when the patients were divided into four groups according to ACPA and ANCA status, differences were present in the outcomes, and the ACPA-positive ANCA-positive group exhibited the lowest cumulative relapse-free survival rate, while no significant difference was present in the relapse between the ANCA-positive ANCA-positive, ACPA-positive ANCA-negative, and ACPA-negative ANCA-positive groups. Finally, the cumulative ILD-free survival rates were comparable between ACPA-positive and ACPA-negative AAV patients. *Conclusions:* The detection of ACPA expression is not uncommon in AAV. However, the presence of ACPA did not influence patients’ basal characteristics and outcomes, suggesting that further exploration of the role of this antibody is needed in patients with AAV.

## 1. Introduction

Anti-citrullinated protein antibodies (ACPAs) are a group of autoantibodies targeting various citrullinated proteins [1]. Citrullination normally occurs during the physiological processes of development and regeneration and is catalysed by peptidyl-arginine deiminases [2]. However, inflammation may trigger a loss of tolerance to citrullinated proteins and promote the pathogenicity of ACPAs, resulting in the continuous generation and maintenance of ACPAs in several autoimmune diseases, such as rheumatoid arthritis (RA) and systemic lupus erythematosus [3,4,5]. Given the exceptionally high specificity of ACPA in RA, ACPA expression was added to the 2010 classification criteria for RA, which was not included in the former 1987 criteria [6,7]. Furthermore, it has been demonstrated that a high ACPA titre could predict an aggressive progression of RA during follow-up [8]. In addition to the clinical significance of ACPAs in the classification and estimation of articular damage in RA, ACPA positivity has been considered to increase the risk of extra-articular manifestations, such as interstitial lung disease (ILD) and cardiovascular disease [9,10].

Antineutrophil cytoplasmic antibody (ANCA)-associated vasculitis (AAV) is a type of small vessel vasculitis, similar to immune complex vasculitis [11]. Depending on their clinical, biochemical, radiological, and histological features, three subtypes of AAV can be distinguished: eosinophilic granulomatosis with polyangiitis (EGPA), granulomatosis with polyangiitis (GPA), and microscopic polyangiitis (MPA) [11,12]. AAV and ACPAs are both associated with the formation of neutrophil extracellular traps (NETs) [13,14]. In fact, previous studies have reported that the formation of NETs is directly related to both the production of ANCAs and AAV activity [15,16]. Another study has demonstrated the anti-inflammatory properties of a therapeutic ACPA binding to specific sites of citrullines, which resulted in an alleviation of the inflammatory burden in an animal model of inflammatory arthritis [17].

Based on the current available evidence, it can be reasonably speculated that ACPAs may occur in AAV patients and could accelerate AAV activity through the formation of NETs. However, to date, no study has elucidated the clinical significance of ACPAs in patients with AAV. Hence, in this study, we measured the rate of ACPA positivity in patients with AAV and investigated the association of ACPAs with their clinical features and outcomes during follow-up.

## 2. Materials and Method

### 2.1. Selection of Study Subjects

We retrospectively reviewed the medical records of 244 patients with AAV. All patients were classified as having AAV according to the European Medicine Agency AAV algorithm proposed in 2007 and the revised International Chapel Hill Consensus Conference Nomenclature of Vasculitides issued in 2012. They were initially diagnosed at the Division of Rheumatology in the Department of Internal Medicine at Yonsei University College of Medicine in Severance Hospital from October 2000 to December 2020. All patients had well-documented medical records, which were used to review their clinical features and laboratory test results, including those from tests for ANCA positivity, and assess the initial Birmingham Vasculitis Activity Score (BVAS) and Five-Factor Score (FFS). Patients who had been followed up for fewer than 3 months and had medical conditions such as malignancies, serious infectious diseases, or other types of systemic vasculitides at diagnosis and RA, were excluded [7].

Of the initially considered 244 AAV patients, 75 were excluded from the study, as ACPA was not tested at their disease diagnosis. Of the remaining 169 AAV patients, one patient was excluded from the study because this patient could be classified as having an overlapping syndrome of both AAV and RA. The clinical characteristics of this patient were positive myeloperoxidase-ANCA, ground-glass opacity observed in chest imaging, and suggested renal vasculitis showing as haematuria and proteinuria. Pauci-immune rapidly progressive glomerulonephritis was demonstrated in kidney biopsy. Therefore, the patient’s diagnosis was concluded as MPA [11]. Nevertheless, as this patient also fulfilled the 2010 classification criteria for RA due to the presence of joint pain and arthritis documented through a whole-body bone scan, high serum concentration of ACPAs, and elevated ESR and CRP, this patient was excluded upon study conception [7].

**Figure 1:**
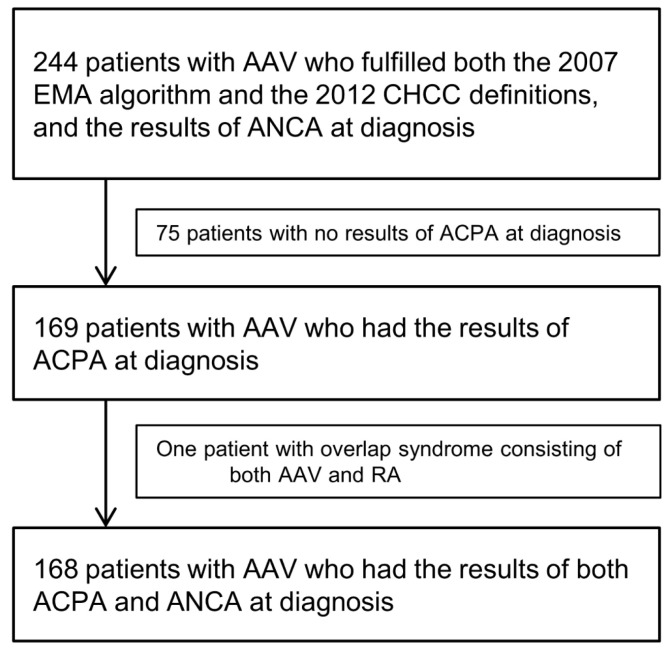
Flow diagram of selecting 168 patients. AAV: Antineutrophil cytoplasmic antibody-associated vasculitis; EMA: European medicines agency; CHCC: Chapel Hill Consensus Conference; ANCA: Antineutrophil cytoplasmic antibody; ACPA: Anti-citrullinated protein antibody; RA: Rheumatoid arthritis.

Ultimately, 168 AAV patients with both ACPA and ANCA results available were included in the study (Figure 1). The present study was approved by the institutional review board (IRB) of Severance Hospital (Seoul, Korea, IRB No. 4-2020-1071). Given the retrospective design of the study and the use of anonymised patient data, the requirement for written informed consent was waived.

### 2.2. Variables Assessment at Diagnosis

Age, gender, and smoking history were recorded as demographic data. The number of ACPA-positive patients was counted and the associated titres measured using enzyme-linked immunosorbent assay were also reviewed. Information on AAV subtypes, ANCA positivity, BVAS-based items, and AAV-specific indices, including BVAS and FFS, was obtained [18,19,20]. The presence of concurrent comorbidities and the results of the acute phase reactants of erythrocyte sedimentation rate (ESR) and C-reactive protein (CRP) were also assessed.

### 2.3. Outcomes Evaluated during the Follow-up

Occurrences of all-cause mortality, relapse, and end-stage renal disease (ESRD), as well as ILD were evaluated as outcomes. The death-based follow-up period was defined as the interval from the day of AAV diagnosis to the day of death for deceased patients and the day of the last visit for surviving patients. The relapse-based follow-up period was defined as the interval from the time of diagnosis to the time of relapse for patients experiencing relapse and the day of the last visit for those who did not experience relapse, which was prespecified in the European League Against Rheumatism recommendations [21]. In addition, the ESRD-based follow-up period was defined as the interval from the time of diagnosis to the initiation of renal replacement treatment for patients with ESRD and the day of the last visit for those without ESRD. Finally, the ILD-based follow-up period was defined as the interval from the time of diagnosis to the time of ILD development for patients with ILD and the day of the last visit for those without ILD; the follow-up period for patients with ILD at diagnosis was considered as zero. The number of patients who received glucocorticoids and the immunosuppressive drugs cyclophosphamide, rituximab, mycophenolate mofetil, azathioprine, tacrolimus, and methotrexate were counted.

### 2.4. Statistical Analyses

All statistical analyses were performed using IBM SPSS Statistics for Windows, version 25 (IBM Corp., Armonk, NY, USA). Continuous variables were expressed as a mean with standard deviation, whereas categorical variables were expressed as numbers (percentages). Significant differences among two continuous variables were assessed using the Student’s *t*-test and categorical variables were analysed using chi-squared and Fisher’s exact tests. A Pearson’s correlation analysis was carried out to calculate the correlation coefficient between two variables. Overall and pairwise comparisons of the cumulative survival rates of the patient groups were performed by a Kaplan–Meier survival analysis with a log-rank test, as appropriate. Statistical significance was set at *p* < 0.05.

## 3. Results

### 3.1. Baseline Characteristics

In terms of the variables evaluated at diagnosis, the mean age of the 168 AAV patients was 58.6 years, and 33.3% were male. The detection rate of ACPAs in AAV patients was 8.9%, and 135 patients (80.4%) were positive for ANCAs. The most common BVAS item was pulmonary involvement (64.9%), followed by renal involvement (58.9%). The mean BVAS and FFS were 13.3 and 1.3, respectively. Sixty-seven patients (39.9%) had hypertension and 48 patients (28.6%) presented with interstitial lung disease. For the outcomes assessed during follow-up, 16 patients (9.5%) died, 55 patients (32.7%) experienced relapse, and 28 patients (16.7%) progressed to ESRD. Glucocorticoids, azathioprine, and cyclophosphamide were administered to 156 (92.9%), 93 (55.4%), and 88 patients (52.4%), respectively (Table 1).

### 3.2. Differences between 153 ACPA-Negative and 15 ACPA-Positive AAV Patients

Among the variables measured at diagnosis, there were no significant differences in demographic data between ACPA-negative and ACPA-positive AAV patients. Similarly, the proportion of AAV subtypes, the ANCA detection rates, the number of clinical manifestations based on BVAS, the AAV-specific indices, the type of comorbidities, and the results of laboratory tests did not differ between these two groups. Furthermore, during the follow-up, no significant differences in administered medications were found between the two groups (Table 1). Additionally, the titre of ACPAs was not significantly correlated with BVAS (r = −0.066, *p* = 0.394), FFS (r = −0.111, *p* = 0.153), ESR (r = −0.137, *p* = 0.076), or CRP (r = −0.084, *p* = 0.282).

### 3.3. Comparison of Overall, Relapse-Free, and ESRD-Free Survival Rates between ACPA-Negative and ACPA-Positive AAV Patients

**Figure 2:**
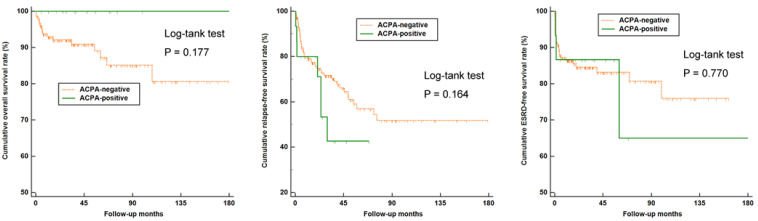
Comparison of overall, relapse-free, and ESRD-free survival rates between ACPA-negative (*n* = 153) and ACPA-positive (*n* = 15) AAV patients. ESRD: End-stage renal disease; ACPA: Anti-citrullinated protein antibody; AAV; Antineutrophil cytoplasmic antibody-associated vasculitis.

**Figure 3:**
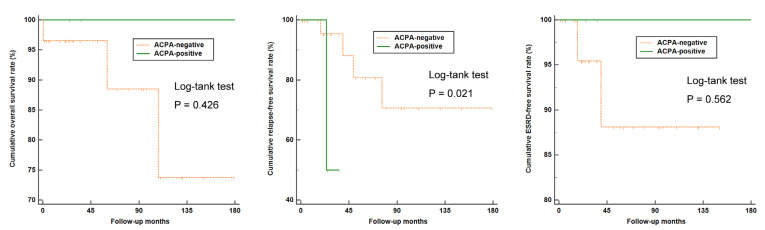
Overall, relapse-free, and ESRD-free survival rates between ACPA-negative and ACPA-positive patients in ANCA-negative AAV (*n* = 33) patients. ESRD: End-stage renal disease; ACPA: Anti-citrullinated protein antibody; ANCA: Antineutrophil cytoplasmic antibody; AAV: Antineutrophil cytoplasmic antibody-associated vasculitis.

There were no significant differences in overall, relapse-free, and ESRD-free survival rates between ACPA-negative and ACPA-positive AAV patients (Figure 2). In addition, we compared the overall, relapse-free, and ESRD-free survival rates between ACPA-negative and ACPA-positive patients among 33 ANCA-negative AAV patients. In these patients, those with ACPA positivity exhibited a significantly higher rate of experiencing disease relapse compared to the ACPA-negative group (*p* = 0.021) (Figure 3).

### 3.4. Disease Relapse Rate among Patients with and without ANCA

It was previously demonstrated that ANCA is a predictor of disease relapse in patients with AAV [22]. In comparing the cumulative relapse-free survival rates of patients with and without ANCA, we found that ANCA-positive AAV patients exhibited a significantly lower cumulative relapse-free survival rate than ANCA-negative AAV patients, consistent with the existing literature (*p* = 0.021) (Figure S1).

### 3.5. Comparison of the Overall, Relapse-Free, and ESRD-Free Survival Rates According to ACPA and ANCA Detection

**Figure 4:**
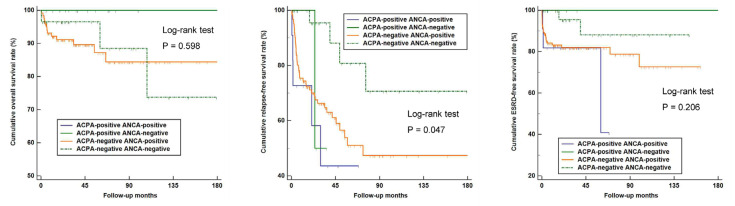
The overall, relapse-free, and ESRD-free survival rates according to the presence of ACPA and ANCA. ESRD: End-stage renal disease; ACPA: Anti-citrullinated protein antibody; ANCA: Antineutrophil cytoplasmic antibody; AAV; Antineutrophil cytoplasmic antibody-associated vasculitis.

Next, AAV patients were divided into four groups according to the results of the ACPA and ANCA detection: 11, 4, 124, and 29 patients were assigned to the ACPA-positive ANCA-positive, ACPA-positive ANCA-negative, ACPA-negative ANCA-positive, and ACPA-negative ANCA-negative groups, respectively. There were no significant differences in the overall and ESRD-free survival rates among AAV patients of the four groups. On the other hand, a difference was noted in the rate of relapse among the groups, and ACPA-positive ANCA-positive AAV patients showed the lowest cumulative relapse-free survival rate (*p* = 0.047) (Figure 4). However, there was no significant difference in the relapse rates of between the ANCA-positive ANCA-positive, ACPA-positive ANCA-negative, and ACPA-negative ANCA-positive groups.

### 3.6. Occurrence of ILD According to ACPA Positivity

**Figure 5:**
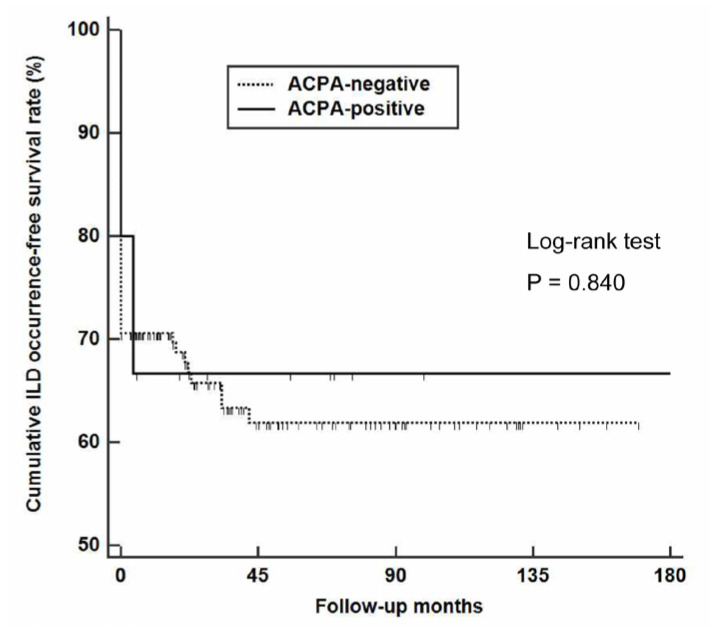
De novo occurrence of ILD in AAV patients according to ACPA status. ILD: Interstitial lung disease; AAV: Antineutrophil cytoplasmic antibody-associated vasculitis; ACPA: Anti-citrullinated protein antibody.

To evaluate the influence of ACPA expression on the development of ILD during follow-up, we searched for newly developed ILD in AAV patients and found it in two ACPA-positive and eight ACPA-negative AAV patients. When applying the Kaplan–Meier survival analysis, we found no difference in the cumulative ILD-free survival rates of ACPA-positive and ACPA-negative AAV patients (*p* = 0.840) (Figure 5).

## 4. Discussion

In this study, we first assessed the ACPA detection rate in AAV patients and investigated the association of ACPAs through both concomitant AAV activity and outcomes during follow-up. These analyses yielded the following findings: (i) ACPAs were detected in 8.9% of AAV patients; (ii) neither ACPA positivity nor ACPA levels were associated with concomitant AAV-specific indices or acute phase reactants; (iii) ACPA-negative and ACPA-positive AAV patients did not show any differences in terms of the incidence of outcomes; (iv) the additional prognostic role of having both ACPA and ANCA was not evident, as the rates of disease relapse were comparable among the ACPA-positive ANCA-positive, ACPA-positive ANCA-negative, and ACPA-negative ANCA-positive groups.

ACPA is generally considered a highly specific antibody in patients with RA or individuals at risk for developing RA [23]. Intriguingly, there were studies that analysed the implications of positive ACPAs in patients with autoimmune diseases. A previous study by Skare et al. [24] investigated ACPA positivity rates in systemic lupus erythematosus (SLE) and reported that 13.7% of patients—which was relatively high—with SLE had positive ACPA. However, similar to the results of our study, no differences in the patients’ clinical profiles were revealed. These observations were replicated in an analysis of two well-defined European cohorts of patients with SLE that showed similar American College of Rheumatology-defined phenotypes in those with and without ACPA [25], while the study by Ziegelasch reported ACPA positive rates as 5.4–6.8%. On the other hand, an Italian study that investigated ACPA in patients with primary Sjögren’s syndrome demonstrated ACPA positivity of 9.9%, revealing an association with prevalent synovitis [26]. Our results found that the detection rate of ACPA in AAV was 8.9%, which appears to be higher than is detected in the normal population [27]. Altogether, these findings indicate that ACPA detection may not be an exclusive laboratory feature in patients with RA and may be detected more frequently in patients with autoimmune diseases.

In patients with RA, the presence of ACPAs and ACPA titre have been considered to be associated with the development of ILD [9,28]. In particular, the expression of anti-citrullinated enolase peptide-1 was previously reported to be closely associated with ILD and suggested as a biomarker for predicting its development in patients with RA [29]. Thus, we hypothesized that ACPA-positive AAV patients might exhibit a higher incidence rate of ILD than ACPA-negative AAV patients either at diagnosis or during the disease course. However, no significant difference was found in the rate of ILD at diagnosis and de novo occurrences of ILD on follow-up according to ACPA detection. Moreover, the frequency of pulmonary manifestations at diagnosis did not differ between these two groups. Therefore, it could be concluded that ACPA expression is likely not associated with ILD in patients with AAV, unlike with RA.

A comparison of the baseline characteristics of ACPA-positive and negative patients showed that there was no difference in the investigated variables at disease diagnosis. Furthermore, it was demonstrated that the outcomes of all-cause mortality, relapse, and end-stage renal disease were similar between ACPA-positive and negative patients, suggesting that the clinical value of this antibody may be relatively limited. It is unknown whether having ACPA positivity increases relapse in AAV; however, a previous investigation showed that patients double-seropositive for ANCA and the anti-glomerular basement membrane antibody have a distinguishing clinical outcome [30], raising a possibility that the coexistence of both ANCA and ACPA might be also associated with an increased risk of disease relapse, based on the following reasons: (i) ANCA positivity is a well-known factor of relapsing disease in AAV patients [22] and (ii) a previous observation from the RETRO study demonstrated that RA patients with ACPA positivity had a higher risk of relapse [31]. Supporting this result, we found that AAV patients with ACPA positivity had a significantly higher rate of disease relapse in a subgroup analysis of those with ANCA negativity, as shown in Figure 3. In our analysis, although the patient outcomes of all-cause mortality and ESRD were comparable, there was a difference in the disease relapse rate when patients were categorized into four groups according to their ACPA and ANCA detection status. In particular, patients with ACPA and ANCA double positivity appeared to have the lowest cumulative relapse-free survival during the follow-up, while statistical significance was not evident compared to the ACPA-positive ANCA-negative and ACPA-negative ANCA-positive groups. As the number of patients in each subgroup was very small and while a cautious interpretation should be given, it seems evident that future studies are warranted to investigate the influence of having ACPA positivity in addition to ANCAs in estimating patient outcomes.

One merit of our study is that we first investigated the clinical significance of ACPA in a single-centre cohort of AAV patients with the same ethnic and geographical features. However, our study also has several limitations. First, this study was conducted in a single centre and because ACPA testing is not routinely recommended in AAV, a selection bias is present. Second, even though the medications administered were found comparable in the patients, treatment strategies could not be adjusted as this is a retrospective study. Third, the pathologic mechanism resulting in the expression of ACPA in AAV patients could not be determined in this study. Future prospective and multi-centre studies in AAV patients will validate our results and demonstrate more reliable information on the impact of ACPA in AAV.

## 5. Conclusions

In conclusion, ACPA expression is not uncommon in AAV. However, its influence in patient baseline characteristics and clinical outcomes were not evident. Moreover, the concurrent expression of both ACPA and ANCA also did not affect disease relapse. Additional research seems to be warranted to elucidate the clinical significance of ACPA positivity in patients with AAV.

## Figures and Tables

**Table 1:** Characteristics at diagnosis, outcomes, and medications during follow-up in 168 patients with AAV.

Variables	Total Patients (*N* = 168)	ACPA-Negative AAV Patients (*N* = 153)	ACPA-Positive AAV Patients (*N* = 15)	*p*-Value
At the time of diagnosis				
**Demographic data**				
Age (years)	58.6 (13.2)	59.0 (13.2)	54.1 (12.9)	0.165
Male gender (*N*, (%))	56 (33.3)	53 (34.6)	3 (20.0)	0.390
Smoking history (*N*, (%))	5 (3.0)	5 (3.3)	0 (0)	1.000
**ACPA positivity (*N*, (%))**	15 (8.9)	0 (0.0)	15 (100.0)	<0.001
**AAV subtypes (*N*, (%))**				0.960
MPA	95 (56.5)	86 (56.2)	9 (60.0)	
GPA	36 (21.4)	33 (21.6)	3 (20.0)	
EGPA	37 (22.0)	34 (22.2)	3 (20.0)	
**ANCA positivity (*N*, (%))**				
MPO-ANCA (or P-ANCA) positivity	123 (73.2)	112 (73.2)	11 (73.3)	1.000
PR3-ANCA (or C-ANCA) positivity	20 (11.9)	18 (11.8)	2 (13.3)	0.694
Both	8 (4.8)	6 (3.9)	2 (13.3)	0.152
ANCA negative	33 (19.6)	29 (19.0)	4 (26.7)	0.498
**Clinical manifestations based on BVAS items (*N*, (%))**				
General	70 (41.7)	64 (41.8)	6 (40.0)	0.891
Articular	15 (8.9)	12 (7.8)	3 (20.0)	0.136
Cutaneous	35 (20.8)	30 (19.6)	5 (33.3)	0.212
Mucous and ocular	4 (2.4)	4 (2.6)	0 (0)	1.000
Otorhinolaryngologic	80 (47.6)	75 (49.0)	5 (33.3)	0.288
Pulmonary	109 (64.9)	101 (66.0)	8 (53.3)	0.326
Cardiovascular	35 (20.8)	33 (21.6)	2 (13.3)	0.739
Gastrointestinal	8 (4.8)	8 (5.2)	0 (0)	1.000
Renal	99 (58.9)	91 (59.5)	8 (53.3)	0.644
Nervous systemic	63 (37.5)	56 (36.6)	7 (46.7)	0.442
**AAV-specific indices**				
BVAS	13.3 (7.4)	13.4 (7.4)	12.0 (7.9)	0.491
FFS	1.3 (1.0)	1.3 (1.0)	1.1 (1.1)	0.299
**Comorbidities (*N*, (%))**				
Diabetes Mellitus	44 (26.2)	42 (27.5)	2 (13.3)	0.358
Hypertension	67 (39.9)	63 (41.2)	4 (26.7)	0.408
Dyslipidaemia	28 (16.7)	25 (16.3)	3 (20.0)	0.718
Interstitial lung disease	48 (28.6)	45 (29.4)	3 (20.0)	0.559
**Acute phase reactants**				
ESR (mm/hr)	59.1 (38.8)	60.9 (38.6)	40.8 (36.6)	0.055
CRP (mg/L)	38.5 (51.4)	39.8 (51.9)	25.2 (45.4)	0.312
**Medications administered during the follow-up period (*N*, (%))**				
Glucocorticoids	156 (92.9)	141 (92.2)	15 (100)	0.604
Cyclophosphamide	88 (52.4)	82 (53.6)	6 (40.0)	0.314
Rituximab	25 (14.9)	23 (15.0)	2 (13.3)	1.000
Mycophenolate mofetil	21 (12.5)	18 (11.8)	3 (20.0)	0.406
Azathioprine	93 (55.4)	87 (56.9)	6 (40.0)	0.210
Tacrolimus	13 (7.7)	11 (7.2)	2 (13.3)	0.327
Methotrexate	15 (8.9)	12 (7.8)	3 (20.0)	0.136

Values are expressed as a mean (standard deviation) or *N* (%). ANCA: antineutrophil cytoplasmic antibody; AAV: ANCA-associated vasculitis; ACPA: anti-citrullinated peptide antibody; MPA: microscopic polyangiitis; GPA: granulomatosis with polyangiitis; EGPA: eosinophilic granulomatosis with polyangiitis; MPO: myeloperoxidase; P: perinuclear; PR3: proteinase 3; C: cytoplasmic; BVAS: Birmingham Vasculitis Activity Score; FFS: Five-Factor Score; ESR: erythrocyte sedimentation rate; CRP: C-reactive protein.

## Data Availability

The datasets generated during and/or analysed during the current study are available from the corresponding author on reasonable request.

## Supplementary Materials

The following supporting information can be downloaded at: https://www.mdpi.com/article/10.3390/medicina58040558/s1. Supplementary Figure S1: Comparison of relapse-free survival rate in ANCA-positive and ANCA-negative AAV patients.
