# Supplementary figures and images for: Anti-Citrullinated Peptide Antibody Expression and Its Association with Clinical Features and Outcomes in Patients with Antineutrophil Cytoplasmic Antibody-Associated Vasculitis
Source: Medicina (Kaunas). 2022 Apr 18;58(4):558. doi: 10.3390/medicina58040558 (PMC9025032; doi:10.3390/medicina58040558)

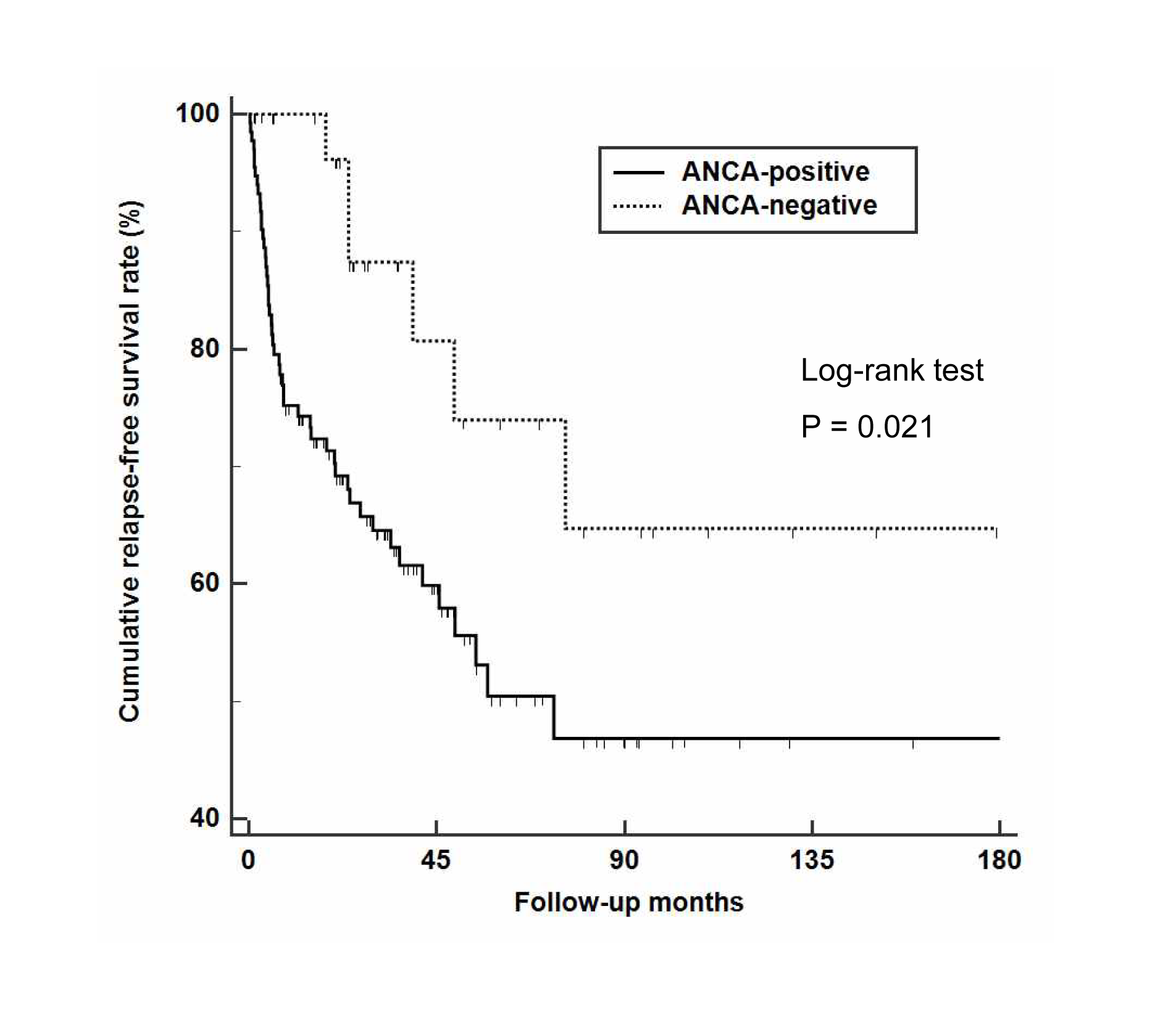

Supplement: Supplementary file 1 [file medicina-58-00558-s001.zip › Supplementary Figure S1.tif]
